# Supplementary material for: Group contracts and sustainability: Experimental evidence from smallholder seed production
Source: PLoS One. 2021 Aug 18;16(8):e0255176. doi: 10.1371/journal.pone.0255176 (PMC8372911; doi:10.1371/journal.pone.0255176)

**Appendix tables and figures:**

**Table A1:** Socio-economic characteristics of seed producers and organizers

| **Characteristics** | | **Mean** | **Min** | **Max** |
| --- | --- | --- | --- | --- |
| Seed producers | Age | 45.61 (11.25) | 2.00 | 84.00 |
|  | Education | 7.70  (4.57) | 0.00 | 18.00 |
|  | Land owned | 4.79  (3.48) | 0.00 | 25.00 |
|  | Proportion of land under seed production | 0.79  (0.28) | 0.00 | 1.00 |
|  | Years of experience in seed production | 16.85  (83.75) | 0.00 | 20.00 |
|  | Price per kg (hybrid) | 63.25  (11.77) | - | - |
|  | Price per kg (OPV) | 16.28  (8.62) | - | - |
| Seed organizes | Age | 41.01  (7.77) | 27.00 | 58 |
|  | Education (years of schooling) | 11.15  (2.75) | 0.00 | 15 |
|  | Experience (number of years) | 10  (5.09) | 2.00 | 24 |
|  | Service charges (Rs. per kg of seed) | 1.49  (2.0) | 0.25 | 4.5 |
|  | Area organized under each variety (acres) | 83.68  (98.20) | 5.00 | 1000 |
| *Standard deviations in the parenthesis* | | | | |

**Table A2:** Balance test across the treatments

| **Particulars** | **Kruskal-Wallis –median equality test** | |
| --- | --- | --- |
|  | Chi-square | p-values |
| Age | 6.85 | 0.0769 |
| Education | 6.1 | 0.1073 |
| Land owned | 5.36 | 0.1476 |
| Area under seed production | 7.10 | 0.0702 |
| Years of experience | 29.42 | 0.0001 |
| Type of variety grown | 28.64 | 0.0001 |

**Table** **A3:** Payoff calculation for different contracts

| **Subject** | **Contract type** | **Weather/production risk** | **Quality of seeds produced** | **Payoff function** |
| --- | --- | --- | --- | --- |
| Producer | A | weather risk | No output | Endowment – effort cost |
|  |  | No weather risk | Sub-Standard | Endowment – effort cost + grain price |
|  |  |  | Standard | Endowment – effort cost + price * quantity |
|  |  |  | Excellent | Endowment – effort cost + (price +10% price) * quantity |
|  | B and C | weather risk | No output | Endowment – effort cost |
|  |  | No weather risk | Substandard | Endowment – effort cost + membership cost +cost reduction on input + grain price |
|  |  |  | Standard | Endowment – effort cost + membership cost + cost reduction on input + price * quantity |
|  |  |  | Excellent | Endowment – effort cost + membership cost +cost reduction on input + (price +10% price) * quantity |
| Organizer | A | weather risk | No output | Endowment – effort cost |
|  |  | No weather risk | Substandard | Endowment – effort cost |
|  |  |  | Standard | Endowment – effort cost + commission rate per kg + (price offered by company-price offered by the organizer)*quantity |
|  |  |  | Excellent | Endowment – effort cost + commission rate per kg + (price offered by company-price offered by the organizer)*quantity |
|  | B and C | weather risk | No output | Endowment – effort cost |
|  |  | No weather risk | Substandard | Endowment – effort cost |
|  |  |  | Standard | Endowment – effort cost + commission rate per kg * no. of producers in SPG + (price offered by company-price offered by the organizer)*quantity* no. of members in SPG |
|  |  |  | Excellent | Endowment – effort cost + commission rate per kg * no. of producers in SPG + (price offered by company-price offered by the organizer)*quantity* no. of members in SPG |

| **Table A4:** Experimental outcomes under different contracts | | | | | | |
| --- | --- | --- | --- | --- | --- | --- |
| **Outcomes** | **Contract  A** | | **Contract  B** | | **Contract C** | |
|  | **Hybrid** | **OPV** | **Hybrid** | **OPV** | **Hybrid** | **OPV** |
| Offered price (INR) | 61.35 | 17.93 | 60.28  (1.62) | 16.87  (1.01) | 64.17  (1.00) | 19.58  (0.53) |
|  |  |  |  |  |  |  |
| Final price (INR) | 61.35 | 17.93 | 62.58  (1.53) | 18.31  (0.69) | 66.16  (1.45) | 21.33  (1.14) |
|  |  |  |  |  |  |  |
| Price gain (INR) | - | | 2.29  (1.43) | 1.44  (0.88) | 2.00  (1.09) | 1.75  (1.01) |
|  |  |  |  |  |  |  |
| Length bargaining | - | | 2.92  (0.27) | 2.89  (0.31) | 1.93  (0.90) | 1.95  (0.87) |
|  |  |  |  |  |  |  |
| Producer Effort | 3.82 (1.06) | 4.11  (0.98) | 3.63 (1.14) | 3.76 (1.15) | 4.09  (1.02) | 4.10 (1.04) |
|  |  |  |  |  |  |  |
| Organizer effort | 3.45  (0.58) | 3.25  (0.64) | 3.52  (0.66) | 3.36  (0.59) | - | |
|  |  |  |  |  |  |  |
| Producer earning (INR) | 646  (232) | | 693  (235) | | 529^a^  (290) | |
|  |  |  |  |  |  |  |
| Organizer earning per producer  (INR) | 72  (22) | | 63  (18) | | - | |
| *Standard error in the parenthesis*  *^a^ 35% of total production is rejected in contract C, where as it is 15% and 7% for Contract A and B respectively.* | | | | | | |

**Appendix figures**

**Fig A1**. Price bargaining in contract B
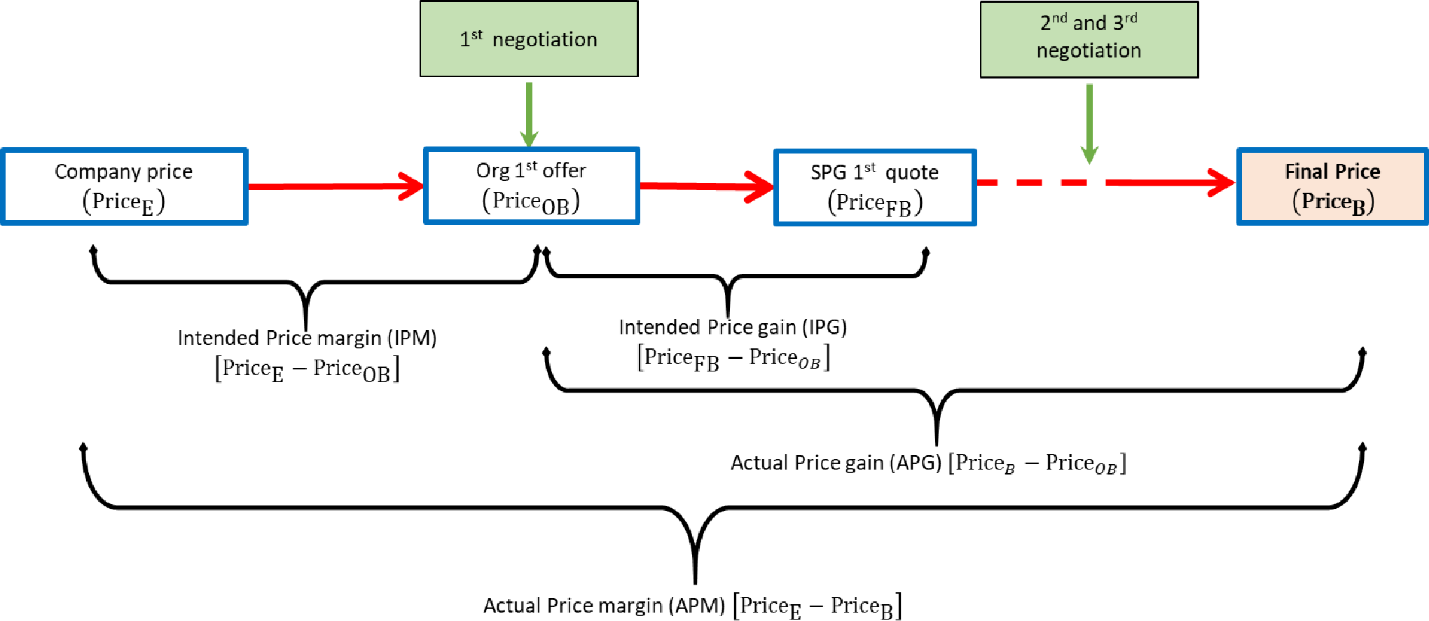


**Fig A2.** See quality producers across contract A, B, and C


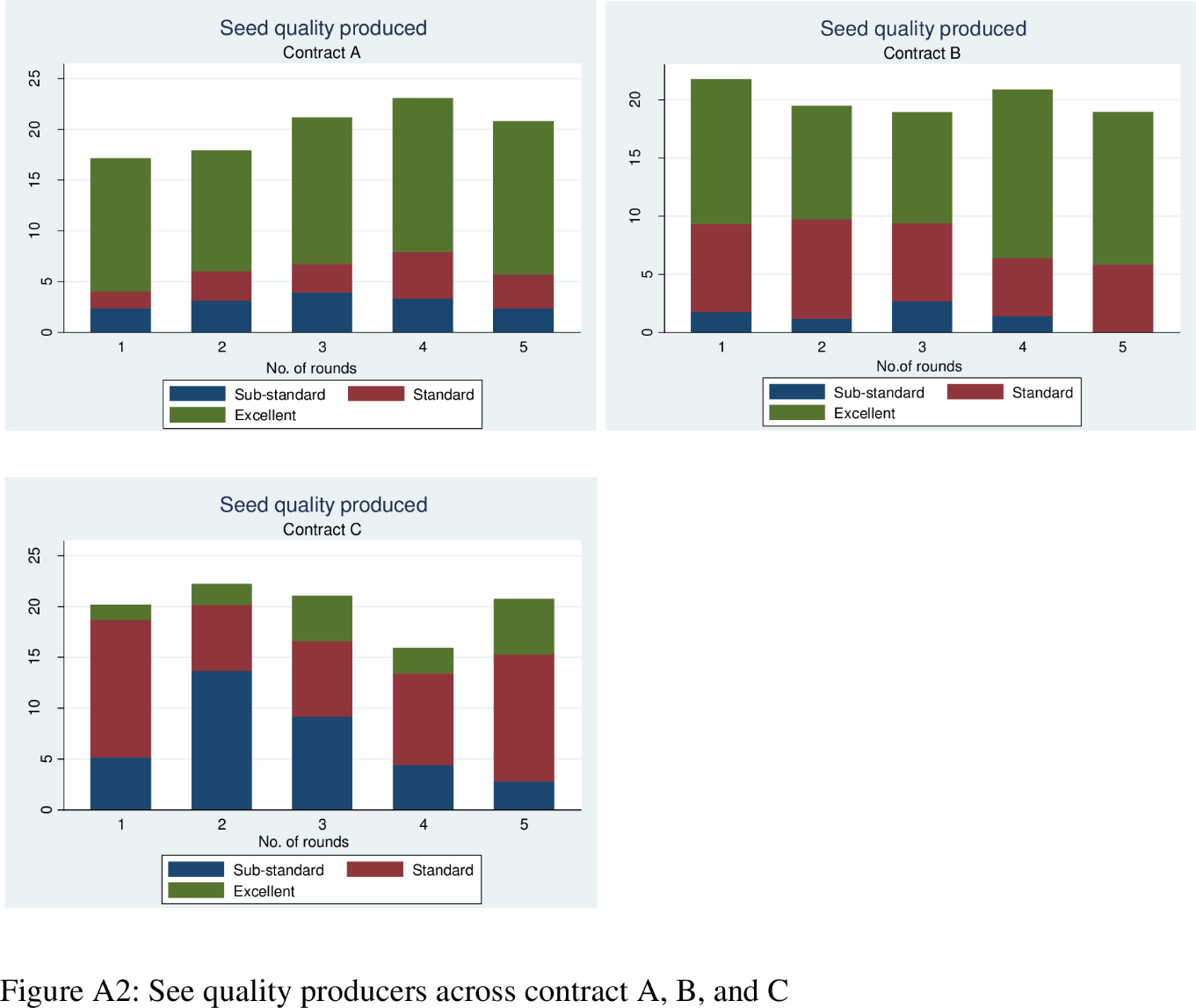

Supplement: S1 Appendix — (DOCX) [file pone.0255176.s001.docx]
