# Supplementary material for: Group contracts and sustainability: Experimental evidence from smallholder seed production
Source: PLoS One. 2021 Aug 18;16(8):e0255176. doi: 10.1371/journal.pone.0255176 (PMC8372911; doi:10.1371/journal.pone.0255176)
Supplement: Attachment — Submitted filename: RESPONSE TO REVIEWERS.docx [file pone.0255176.s002.docx]

Dear Editor,

We are thankful for considering the paper at PLOS ONE. We carefully addressed all comments and also followed the PLOS ONE style requirement as suggested by you. On behalf of all authors, I wanted to thank you and editors for constructive comments and suggestions. You may find our responses against the comments in blue fonts. I hope by addressing those comments the quality of the paper is improved.

Thanks again for considering our paper for publication at PLOS ONE.

Best regards,

Prakashan

**Reviewer comments and responses**

**Comments to the Author**

1. Is the manuscript technically sound, and do the data support the conclusions?

The manuscript must describe a technically sound piece of scientific research with data that supports the conclusions. Experiments must have been conducted rigorously, with appropriate controls, replication, and sample sizes. The conclusions must be drawn appropriately based on the data presented.

Reviewer #1: Partly

Reviewer #2: Yes

Response: Thank you for this positive response. Specific comments of the reviewers are addressed (described in details against the comments)

2. Has the statistical analysis been performed appropriately and rigorously?

Reviewer #1: Yes

Reviewer #2: Yes

Response: Thank you for acknowledging statistical rigor in our analysis.

3. Have the authors made all data underlying the findings in their manuscript fully available?

The [PLOS Data policy](http://www.plosone.org/static/policies.action#sharing) requires authors to make all data underlying the findings described in their manuscript fully available without restriction, with rare exception (please refer to the Data Availability Statement in the manuscript PDF file). The data should be provided as part of the manuscript or its supporting information, or deposited to a public repository. For example, in addition to summary statistics, the data points behind means, medians and variance measures should be available. If there are restrictions on publicly sharing data—e.g. participant privacy or use of data from a third party—those must be specified.

Reviewer #1: Yes

Reviewer #2: Yes

Response: The data is available and if the PLOS ONE has facility to share the data with this manuscript, we are happy to upload in the website. Alternatively, we can make the data available through dataverse.

4. Is the manuscript presented in an intelligible fashion and written in standard English?

PLOS ONE does not copyedit accepted manuscripts, so the language in submitted articles must be clear, correct, and unambiguous. Any typographical or grammatical errors should be corrected at revision, so please note any specific errors here.

Reviewer #1: Yes

Reviewer #2: Yes

Response: We edited the manuscript using a professional editor.

5. Review Comments to the Author

Please use the space provided to explain your answers to the questions above. You may also include additional comments for the author, including concerns about dual publication, research ethics, or publication ethics. (Please upload your review as an attachment if it exceeds 20,000 characters)

Reviewer #1: The paper's approach and overall quality is good. It is researching a remarkable piece of the field, a very actual problem in developing countries. I agree with te conclusions, accept the statistical toolset. I find it a bit long, but the journal's requirements do not limit it so I have to accept.
My advise to do changes as follows:
- a separate literature review should be created, extending the current resources;

Response: As per the reviewer comments we divided the introduction and created a separate literature review session. Following references are added additionally:

- FAO (Food Agric. Org.). Guiding principles for responsible contract farming operations. Rep., Food Agric. Org., Rome: 2012.
- Kirsten J, Sartorius K. Linking agribusiness and small-scale farmers in developing countries: Is there a new role for contract farming? Dev. South. Afr. 2002; 19(4):503–29
- MacDonald JM, Korb P. Agricultural contracting update: contracts in 2008. Econ. Inf. Bull. 72, Econ. Res.Serv., Dep. Agric.,Washington, DC: 2011.
- Minot NW, Swayer B. Contract Farming in Developing Countries: Theory, Practice, and Policy. In: Devaux A, Torero M, Donovan J, Horton, DE, editors. Innovation for inclusive value-chain development: Successes and challenges. Washington, D.C.: International Food Policy Research Institute (IFPRI). 2016.
- Otsuka, K., Nakano, Y., Takahashi, K. Contract farming in developed and developing countries. Annual Review of Resource Economics, 2016; 8: 353–376.

- the limitations of the research should be indicated at the end of conclusions

Response: Thanks for this comment. Limitations of the research is highlighted as per the suggestion.

Refer lines 826 to 839

“We conclude the study highlighting few important limitations, which are of interest for further investigation. In this study, we did not account the free rider problem explicitly. The incentive for a member to free ride can have significance consequences on group performance and quality of the product which can cause net welfare loss for the group as well as for the member who practices free ride. Further, we assumed that the group introduces compliance mechanism when free rider problem exists beyond a threshold level. However, if free rider exists even after these measures, and if so, how do they influence the performance of group is a topic of future investigation. Second, there could be multiple ways that an organizer influences the group formation and its performance. In the present study, the organizers influence is captured only during the formation of group through their presence. But, though the transaction costs of organizers might reduce had they engage in group, but the increased bargaining power of the group will eventually lead to welfare distribution among seed value chain actors, reducing the organizer’s profit. The influence of organizer on group performance under different contracts and incentives to organizers can be explored in future.”

Reviewer #2: This is a very well prepared paper on a topic of international relevance. The paper meets all the scientific requirements to be published in PLOSOne. The literature review, methods and results as well as discussion are very well elaborated.

Response: Thank you for the positive remarks. As per the reviewer 1 suggestion, we added a separate literature review section in the revised manuscript

6. PLOS authors have the option to publish the peer review history of their article ([what does this mean?](https://journals.plos.org/plosone/s/editorial-and-peer-review-process#loc-peer-review-history)). If published, this will include your full peer review and any attached files.


If you choose “no”, your identity will remain anonymous but your review may still be made public.


**Do you want your identity to be public for this peer review?** For information about this choice, including consent withdrawal, please see our [Privacy Policy](https://www.plos.org/privacy-policy).

Reviewer #1: No

Reviewer #2: No

 Response: We respect reviewer’s discretion of being anonymous. We wanted to thank them for their time and comments.

[NOTE: If reviewer comments were submitted as an attachment file, they will be attached to this email and accessible via the submission site. Please log into your account, locate the manuscript record, and check for the action link "View Attachments". If this link does not appear, there are no attachment files.]

While revising your submission, please upload your figure files to the Preflight Analysis and Conversion Engine (PACE) digital diagnostic tool, <https://pacev2.apexcovantage.com/>. PACE helps ensure that figures meet PLOS requirements. To use PACE, you must first register as a user. Registration is free. Then, login and navigate to the UPLOAD tab, where you will find detailed instructions on how to use the tool. If you encounter any issues or have any questions when using PACE, please email PLOS at [figures@plos.org](mailto:figures@plos.org). Please note that Supporting Information files do not need this step.

Response: We will submit all figures through PACE
